# In vitro studies evaluating the activity of imipenem in combination with relebactam against *Pseudomonas aeruginosa*

**DOI:** 10.1186/s12866-019-1522-7

**Published:** 2019-07-04

**Authors:** Katherine Young, Ronald E. Painter, Susan L. Raghoobar, Nichelle N. Hairston, Fred Racine, Douglas Wisniewski, Carl J. Balibar, Artjohn Villafania, Rumin Zhang, Daniel F. Sahm, Timothy Blizzard, Nicholas Murgolo, Milton L. Hammond, Mary R. Motyl

**Affiliations:** 1 Merck & Co., Inc., 2015 Galloping Hill Road MN-410, Kenilworth, NJ 07033 USA; 2 IHMA, Schaumburg, IL USA

**Keywords:** β-Lactamase inhibitor, Carbapenem-resistant, Carbapenemase, Multidrug-resistant, MK-7655, Imipenem/relebactam, Non-susceptible, Antibiotic resistance

## Abstract

**Background:**

The prevalence of antibiotic resistance is increasing, and multidrug-resistant *Pseudomonas aeruginosa* has been identified as a serious threat to human health. The production of β-lactamase is a key mechanism contributing to imipenem resistance in *P. aeruginosa*. Relebactam is a novel β-lactamase inhibitor, active against class A and C β-lactamases, that has been shown to restore imipenem susceptibility. In a series of studies, we assessed the interaction of relebactam with key mechanisms involved in carbapenem resistance in *P. aeruginosa* and to what extent relebactam might overcome imipenem non-susceptibility.

**Results:**

Relebactam demonstrated no intrinsic antibacterial activity against *P. aeruginosa*, had no inoculum effect, and was not subject to efflux. Enzymology studies showed relebactam is a potent (overall inhibition constant: 27 nM), practically irreversible inhibitor of *P. aeruginosa* AmpC. Among *P. aeruginosa* clinical isolates from the SMART global surveillance program (2009, *n* = 993; 2011, *n* = 1702; 2015, *n* = 5953; 2016, *n* = 6165), imipenem susceptibility rates were 68.4% in 2009, 67.4% in 2011, 70.4% in 2015, and 67.3% in 2016. With the addition of 4 μg/mL relebactam, imipenem susceptibility rates increased to 87.6, 86.0, 91.7, and 89.8%, respectively. When all imipenem–non-susceptible isolates were pooled, the addition of 4 μg/mL relebactam reduced the mode imipenem minimum inhibitory concentration (MIC) 8-fold (from 16 μg/mL to 2 μg/mL) among all imipenem–non-susceptible isolates. Of 3747 imipenem–non-susceptible isolates that underwent molecular profiling, 1200 (32%) remained non-susceptible to the combination imipenem/relebactam (IMI/REL); 42% of these encoded class B metallo-β-lactamases, 11% encoded a class A GES enzyme, and no class D enzymes were detected. No relationship was observed between alleles of the chromosomally-encoded *P. aeruginosa* AmpC and IMI/REL MIC.

**Conclusions:**

IMI/REL exhibited potential in the treatment of carbapenem-resistant *P. aeruginosa* infections, with the exception of isolates encoding class B, some GES alleles, and class D carbapenemases.

**Electronic supplementary material:**

The online version of this article (10.1186/s12866-019-1522-7) contains supplementary material, which is available to authorized users.

## Background

*Pseudomonas aeruginosa* is a non-fermenting, aerobic, non-enteric, gram-negative bacterium. Ubiquitous in the environment and globally distributed, it is a leading cause of severe nosocomial infections, particularly in comorbid, immunocompromised, and critically ill patients [1–6]. Antibacterial resistance rates in *P. aeruginosa* are increasing worldwide but show considerable geographic variation [7–20]. Multidrug-resistant (MDR) and extensively drug-resistant (XDR) *P. aeruginosa* strains are becoming increasingly more prevalent and are associated with substantial morbidity and mortality [21, 22].

Carbapenem antibiotics have a very broad antibacterial spectrum and are considered treatments of last resort against increasingly difficult-to-treat drug-resistant pathogens, including *P. aeruginosa* [23, 24]. Imipenem, coadministered with the dehydropeptidase-1 inhibitor cilastatin, remains a mainstay for the empiric and targeted treatment of serious infections, and its use is supported by a wealth of clinical experience [25]. The rise of MDR bacterial pathogens has led to widespread and extensive use of carbapenems. Subsequently, the incidence of carbapenem resistance in *P. aeruginosa* is now increasing worldwide [26–28]. Recent data show about 20% of clinical isolates in the US are non-susceptible (NS) to meropenem, increasing to 65–78% among MDR isolates [29, 30]. Globally, imipenem resistance in *P. aeruginosa* ranges from 16 to 35% [8, 16, 31–35].

In *P. aeruginosa*, non-susceptibility to carbapenems is mediated by a number of resistance mechanisms, which often act synergistically. These mechanisms include reduced permeability of the cell envelope to antibacterial agents through intrinsic characteristics and porin loss/alterations, expression of multidrug efflux pumps, target site alterations, and expression of β-lactamase enzymes [36–42]. Outer membrane porin D (OprD) is the major mechanism by which imipenem transits the *P. aeruginosa* outer membrane in order to enter the periplasmic space and interact with its molecular target (ie, penicillin-binding proteins [PBP]). The two most common imipenem resistance mechanisms in this pathogen are: (a) production of carbapenemases (ie, β-lactamases able to hydrolyze carbapenems) and/or (b) overexpression of AmpC β-lactamase or extended-spectrum β-lactamases in conjunction with decreased OprD permeability, thereby reducing imipenem’s ability to reach PBP [41–44]. Given the clinical relevance of increasing carbapenem resistance in *P. aeruginosa*, new treatments to overcome this problem are urgently needed. One such approach is the restoration of carbapenem susceptibility using β-lactamase inhibitors (BLI).

A novel BLI currently under clinical development is relebactam (MK-7655), which has been shown to restore the in vitro antibacterial activity of imipenem in imipenem-NS strains of gram-negative pathogens, including *P. aeruginosa* [45–47]. Relebactam is a non-β-lactam, small-molecule diazabicyclooctane (DABCO) BLI with activity against class A carbapenemases (such as *Klebsiella pneumoniae* carbapenemase [KPC]) and class C cephalosporinases (including AmpC that can be expressed constitutively from chromosomally-encoded genes) but is inactive against class D carbapenemases like OXA-40 [46–48]. In *P. aeruginosa*, relebactam can restore imipenem susceptibility in otherwise resistant isolates that lack OprD and have either inducible or derepressed AmpC production [45–47]. Recent surveillance studies have demonstrated that the addition of relebactam reduced imipenem minimum inhibitory concentration (MIC) values about 4-fold and increased imipenem susceptibility in all *P. aeruginosa* isolates, including those that were NS and from a lower respiratory tract source [34, 45]. Additional data are needed to elucidate the effects of relebactam against resistance mechanisms in *P. aeruginosa* and to characterize the extent to which relebactam can overcome imipenem non-susceptibility in clinical isolates of this pathogen.

To help answer these questions, we conducted a range of in vitro experiments to evaluate the activity of relebactam against *P. aeruginosa*, including the interaction of relebactam with key mechanisms involved in carbapenem resistance. We also compared the in vitro activities of relebactam plus imipenem versus imipenem alone in several large, world-wide collections of recent clinical *P. aeruginosa* isolates and evaluated the molecular profile of non-susceptibility to the combination.

## Results

### Enzymology studies

**Fig. 1:**
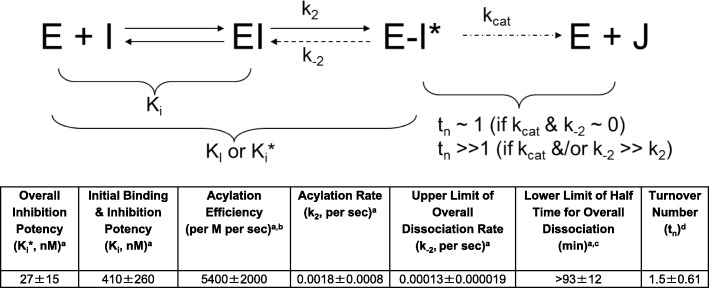
Summary of enzymology studies: kinetic and mechanistic profiling for relebactam against *P. aeruginosa* AmpC. E: AmpC β-lactamase; EI: noncovalent AmpC β-lactamase/relebactam complex; E-I*: acylated AmpC β-lactamase/relebactam complex; I: relebactam; J: hydrolyzed product; k_cat_: turnover rate constant of β-lactamase inhibitor due to solvent water-mediated deacylation; K_i_: dissociation constant for the initial step of noncovalent binding; K_i_*: overall dissociation constant for initial binding and subsequent acylation/deacylation step; K_l_: inactivation constant (if k_− 2_ ~ 0); k_2_: acylation or inactivation rate constant; k_− 2_: intramolecular deacylation or recovery rate constant; t_n_: turnover number (number of compound turnover needed for complete inhibition); k_2_/K_i_: acylation efficiency. ^a^Inhibition kinetic parameter data are based on results from 4 trials. ^b^Ratio of acylation rate constant (k_2_) over K_i_, a measure of acylation efficiency. ^c^0.693/k_− 2_, the minimum estimate of half time for overall inhibitor dissociation. ^d^Relebactam turnover number is based on data from 3 trials and with saturation time of 24 h (data for 2-h saturation time not shown due to similarity with 24-h data)

Results for inhibition kinetic parameters and turnover number are shown in Fig. 1. Relebactam exhibited potent, practically irreversible inhibitory activity to *P. aeruginosa* AmpC with good acylation efficiency; it readily bound to and was acylated by the enzyme. Relebactam also demonstrated long target engagement time and remained bound to the enzyme before slow deacylation and dissociation. Furthermore, the measured turnover number is near unity, approaching that of an irreversible inhibitor.

### Relebactam efflux

The phenotype of the laboratory-derived OprD-deficient mutant strains MB6477 (wild-type efflux-proficient) and MB6476 (multiple efflux-deficient), which were isogenic except for deletion of multiple efflux pumps, was confirmed through antibiograms (Table 1). The MIC values to levofloxacin and chloramphenicol were greatly affected by efflux with a 512-fold and > 128-fold difference in MIC, respectively, between the wild-type and multiple efflux-deficient strains; aztreonam was less affected by efflux, with an 8-fold difference in MIC. These results are expected, given that all of these control antibacterial agents are known resistance-nodulation-cell division (RND) efflux pump substrates [49, 51]. The results of overexpression of each of the 4 RND efflux pumps of *P. aeruginosa* are shown in Table 1; the corresponding 4 mutant isolates were derived from MB6477. Of all antibacterial agents tested, meropenem was most affected by efflux pump overexpression, with a 16-fold increase in MIC in the strain overexpressing MexAB/OprM. The lack of increase in levofloxacin MIC in these pump overexpression mutants is likely due to masking by the underlying *nfxC* mutation in the parental strain, which itself already confers decreased susceptibility to levofloxacin. No influence of efflux, either at baseline levels of RND efflux pump expression or upon overexpression, was seen for imipenem or imipenem/relebactam (IMI/REL), with identical MIC values across the isolate panel. The sole exception was the Δ*nfxB* isolate over-producing MexCD/OprJ; this mutation concurrently results in a down-regulation of AmpC, which in turn results in lower β-lactam MIC values [52].

**Table 1:** Antibiogram of the OprD-deficient *P. aeruginosa* efflux mutant isogenic strain set showing MICs (μg/mL)^a^

Antibacterial agent	MB6476^b^	MB6477^c^	MIC difference^d^	MB6477^c^ Δ*mexR* (overexpresses MexAB)	MB6477^c^ Δ*nfxB* (overexpresses MexCD)	MB6477^c^ Δ*mexL* (overexpresses MexJK)	MB6477^c^ Δ*mexZ* (overexpresses MexXY)
Imipenem	16	16	1-fold	16	4	16	16
IMI/REL^e^	1	1	1-fold	1	0.25	1	1
Ceftazidime^f^	1	1	1-fold	ND	ND	ND	ND
Aztreonam^g^	0.5	4	8-fold	ND	ND	ND	ND
Meropenem^f^	1	1 to 2	1- to 2-fold	16	4	2	2
Chloramphenicol^g^	2	> 256	> 128-fold	ND	ND	ND	ND
Levofloxacin^g^	0.0078	4	512-fold	8	4	4	4

*IMI/REL* imipenem/relebactam, *MIC* minimum inhibitory concentration, *ND* not determined, *OprD* outer membrane porin D, *RND* resistance-nodulation-cell division

^a^MIC values were determined by 2-fold serial broth microdilution

^b^MB6476 *nfxC*, Δ [MexAB-OprM] Δ [MexCD-OprJ] Δ [MexXY] Δ [MexJK-OprLL] Δ [MexHI-OpmD] Δ [OpmH]), spontaneous OprD deletion

^c^MB6477 *nfxC*, wild-type for other efflux pumps, spontaneous OprD deletion

^d^Ratio between the MIC for MB6477 divided by the MIC for MB6476, for the respective antibacterial agent

^e^Imipenem combined with 4 μg/mL of relebactam

^f^Control antibacterial agent known to be an RND efflux pump substrate, with susceptibility known to be adversely affected by efflux pump overexpression [49, 50]

^g^Control antibacterial agent known to be an RND efflux pump substrate, with susceptibility known to be adversely affected even in wild-type *P. aeruginosa* with baseline efflux pump expression [49, 50]

Table 2 shows the BLI concentration required to restore imipenem susceptibility in the wild-type efflux-proficient strain of *P. aeruginosa* (MB6477) and in the isogenic efflux-deficient strain (MB6476), for relebactam and for 3 other representative DABCO BLI (relebactam analogues; see Table 2 for molecular structure), which were chosen for illustrative purposes only. BLI compound 1 and BLI compound 2 were required in greater concentration (> 8-fold and 16-fold, respectively) in the efflux-proficient strain than the efflux-deficient strain to restore imipenem susceptibility, while neither relebactam nor BLI compound 3 required notably higher concentrations in the efflux-proficient versus the efflux-deficient strain (only a 2-fold difference) and therefore did not appear to be subject to efflux.

**Table 2:** BLI concentration required to restore imipenem susceptibility in efflux proficient and deficient *P. aeruginosa*

Compound	Structure	BLI concentration to restore IMI (μg/mL)		Fold differential^b^
		MB6477 efflux-wt^a^	MB6476 efflux-del^a^	
DABCO #1		> 100	12.5	> 8-fold
DABCO #2		50	3.125	16-fold
DABCO #3		12.5	6.25	2-fold
Relebactam		6.25	3.125	2-fold

*BLI* β-lactamase inhibitor, *DABCO* diazabicyclooctane, *IMI* imipenem

^a^MB6477 *nfxC*, wild-type for other efflux pumps, spontaneous OprD deletion; MB6476 *nfxC*, Δ [MexAB-OprM] Δ [MexCD-OprJ] Δ [MexXY] Δ [MexJKL] Δ [MexHI-OpmD] Δ [OpmH])

^b^Differential between the concentrations required to restore susceptibility to imipenem at 4 μg/mL, expressed as the BLI concentration against MB6477 divided by the BLI concentration against MB6476

### Intrinsic antibacterial and inoculum effect

Relebactam demonstrated no intrinsic antibacterial activity when tested alone against 109 clinical isolates of *P. aeruginosa* in concentrations as high as 64 μg/mL. The MIC for relebactam for all isolates in that analysis was > 64 μg/mL.

A change in *P. aeruginosa* inocula to either one log less than or greater than the standard inoculum of 5 × 10^5^ CFU/mL did not have any apparent effect on MIC values of either imipenem or imipenem combined with 4 μg/mL relebactam (Additional file 3: Table S3). This was the case for all 3 isolates examined, whether or not they produced constitutive or inducible AmpC and whether or not they possessed a functional OprD.

### In vitro activity of imipenem versus IMI/REL in clinical isolates

An evaluation of imipenem and IMI/REL susceptibility from the Study for Monitoring Antimicrobial Resistance Trends (SMART) global surveillance program showed little change in their susceptibility profiles from 2009 to 2016. In 2009, 68% of all *P. aeruginosa* isolates (*n* = 993) collected in the SMART surveillance program were susceptible to imipenem, and that proportion increased to 88% with the addition of 4 μg/mL of relebactam to imipenem (Table 3). In 2011, 67% of all *P. aeruginosa* isolates (*n* = 1702) from SMART were imipenem-susceptible, increasing to 86% in combination with 4 μg/mL relebactam (Table 3). In 2015, 70% of all *P. aeruginosa* isolates (*n* = 5953) were susceptible to imipenem, increasing to 92% in combination with 4 μg/mL relebactam. Finally, in 2016, 67% of all *P. aeruginosa* isolates (*n* = 6165) collected in the SMART surveillance program were susceptible to imipenem, and that proportion increased to 90% with the addition of 4 μg/mL of relebactam to imipenem (Table 3). The mode MIC values in the absence and presence of relebactam, respectively, were 1 and 0.25 in 2009 and 2015 and 1 and 0.5 in 2011 and 2016. Correspondingly, the MIC_90_ in the absence and presence of relebactam, respectively, was ≥32 μg/mL and 4 μg/mL in both 2009 and 2011, and was 16 μg/mL and 2 μg/mL in 2015 and 16 μg/mL and 4 μg/mL in 2016. The distribution of MIC values was similar between each of the 4 years (Table 3).

**Table 3:** Distribution of imipenem MIC for *P. aeruginosa* isolates from the SMART global surveillance program^a^

		MIC μg/mL, n (cumulative %)										MIC_90_
		≤0.06	0.125	0.25	0.5	1	2	4	8	16	≥32	
SMART 2009 (*n* = 993 isolates)	IMI	0	4 (0.4)	12 (1.6)	159 (17.6)	308 (48.6)	196 (68.4)	38 (72.2)	26 (74.8)	100 (84.9)	96 (94.6)	≥32
	IMI/REL^b^	0	14 (1.4)	380 (39.7)	306 (70.5)	78 (78.3)	92 (87.6)	46 (92.2)	18 (94.1)	10 (95.1)	10 (96.1)	4
SMART 2011 (*n* = 1702 isolates)	IMI	3 (0.2)	4 (0.4)	15 (1.3)	92 (6.7)	596 (41.7)	437 (67.4)	109 (73.8)	73 (78.1)	162 (87.6)	123 (94.8)	≥32
	IMI/REL^b^	5 (0.3)	9 (0.8)	216 (13.5)	883 (65.4)	207 (77.6)	143 (86.0)	108 (92.3)	37 (94.5)	13 (95.2)	13 (96.0)	4
SMART 2015 (*n* = 5953 isolates)	IMI	ND^c^	ND^c^	ND^c^	1035 (17.4)^d^	2617 (61.3)	540 (70.4)	246 (74.6)	579 (84.3)	565 (93.8)	371 (100.0)	16
	IMI/REL^b^	58 (1.0)	214 (4.6)	2120 (41.7)	1934 (74.2)	587 (84.0)	453 (91.7)	152 (94.2)	111 (96.1)	53 (97.0)	181 (100.0)	2
SMART 2016 (*n* = 6165 isolates)	IMI	ND^c^	ND^c^	ND^c^	925 (15.0)^d^	2593 (57.1)	634 (67.3)	278 (71.9)	567 (81.1)	671 (91.9)	497 (100.0)	16
	IMI/REL^b^	49 (0.8)	169 (3.5)	1875 (33.9)	2299 (71.2)	564 (80.4)	586 (89.9)	154 (92.4)	92 (93.9)	68 (95.0)	309 (100.0)	4

*IMI* imipenem, *IMI/REL* imipenem/relebactam, *MIC* minimum inhibitory concentration, *ND* not determined

^a^Includes SMART surveillance data through October 2018

^b^Imipenem combined with 4 μg/mL of relebactam

^c^This MIC value was not tested

^d^This value is for ≥0.5 μg/mL, since MIC values < 0.5 μg/mL were not tested

**Fig. 2:**
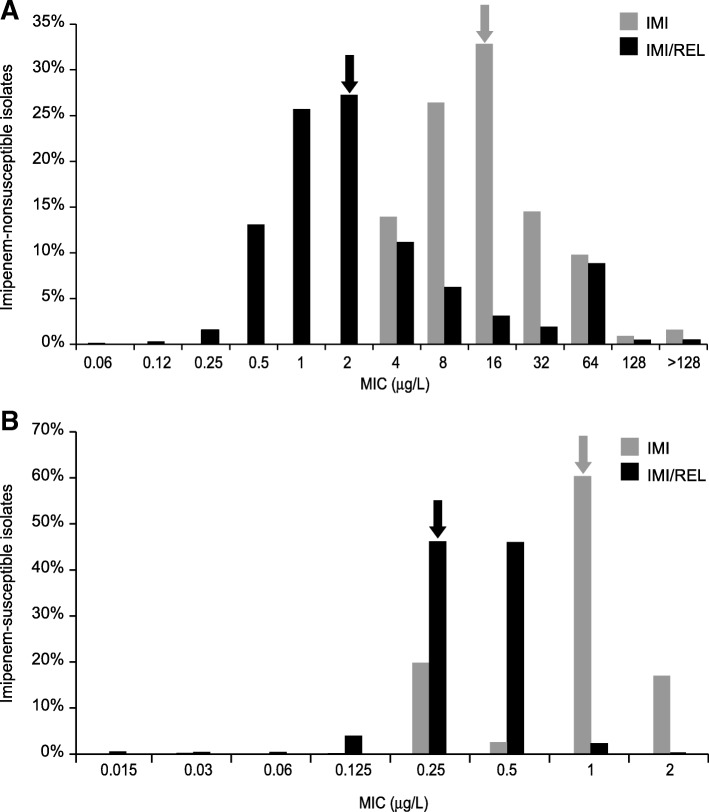
Shift in imipenem MIC values for **a** imipenem-NS (*n* = 4650) and **b** imipenem-susceptible (*n* = 9609) isolates.^a^ Arrows indicate the mode MIC values for imipenem alone and imipenem combined with 4 μg/mL of relebactam. IMI: imipenem; IMI/REL: imipenem/relebactam; MIC: minimum inhibitory concentration; NS: non-susceptible. ^a^Excludes SMART surveillance data from China and India (2015 and 2016) and Vietnam (2015) due to late inclusion of the data in the analysis

At the time of analysis of the shift in imipenem MIC values, there were 4650 imipenem-NS isolates pooled from all sources (Fig. 2a) and 9609 imipenem-susceptible isolates from SMART 2009, 2011, 2015, and 2016 (Fig. 2b). For the imipenem-NS isolates, the MIC range was 4 to > 128 μg/mL for imipenem alone and 0.06 to > 128 μg/mL for IMI/REL; the mode MIC values were 16 μg/mL and 2 μg/mL, respectively, equaling an 8-fold shift. Conversely, in imipenem-susceptible isolates, the MIC range (mode) was 0.03 to 2 μg/mL (1 μg/mL) with imipenem alone and 0.015 to 2 μg/mL (0.25 μg/mL) with IMI/REL, for a 4-fold shift in mode MIC.

### Molecular characterization of non-susceptibility to IMI/REL in clinical isolates

In total, 3747 imipenem-NS isolates underwent molecular profiling for β-lactamases, of which 1200 (32%) were not restored to imipenem susceptibility in the presence of relebactam at 4 μg/mL. In general, the higher the imipenem MIC value of isolates that were not susceptible to the combination of imipenem plus relebactam, the more likely they were to encode class B β-lactamases (ie, metallo-β-lactamases [MBLs]): at an IMI/REL MIC of 4 μg/mL only 3% of isolates contained a gene encoding an MBL, but this proportion increased with each doubling of the MIC, reaching 92% at 64 μg/mL (Table 4). In fact, a gene encoding an MBL was found in 506/1200 (42%) of all isolates NS to the combination (Table 4). In comparison, 222/1200 (19%) of all isolates NS to the combination encoded a class A enzyme and a *Pseudomonas*-derived cephalosporinase (PDC) allele only. A total of 133/1200 (11%) isolates encoded a class A GES enzyme in addition to PDC, and was more commonly found in isolates with IMI/REL MICs of 8 to 32 μg/mL. The class A GES family of β-lactamases in *P. aeruginosa* was observed worldwide, but was most common in Latin America (*n* = 57) and Europe (*n* = 51); other locations included Africa (*n* = 20), the Middle East (*n* = 12), the South Pacific (*n* = 5), Asia (*n* = 4), and North America (*n* = 3). Both ESBL^(e)^ and carbapenemase^(c)^ alleles of GES were found in susceptible isolates (GES-1^e^, −2^c^, −5^c^, −7^e^, −9^e^, and − 29) as well as those which were NS (GES-2^c^, −5^c^, −7^e^, −9^e^, −19^e^, −20^c^, − 26). A total of 472/1200 (39%) encoded AmpC only (all of which encoded PDC, and 2 additionally encoded a plasmid-mediated AmpC). Of note, the proportion of isolates encoding only PDC decreased with imipenem MIC doubling, an inverse relationship with respect to those isolates encoding MBLs. Class D enzymes were not detected in any of the SMART isolates that were NS to IMI/REL.

**Table 4:** Summary of *P. aeruginosa* isolates from SMART (*N* = 1200) NS to IMI/REL^a,b^

MIC^c^ (μg/mL)	N	Class A					Class B	Class C
		PER	GES	VEB	Other ESBL	KPC	Any MBL	AmpC only^d^
4	313	12 (4%)	9 (3%)	18 (6%)	8 (3%)	3 (1%)	9 (3%)	254 (81%)
8	239	4 (2%)	49 (21%)	7 (3%)	3 (1%)	5 (2%)	29 (12%)	142 (59%)
16	128	–	37 (29%)	1 (1%)	3 (2%)	9 (7%)	31 (24%)	47 (37%)
32	84	–	26 (31%)	–	–	6 (7%)	36 (43%)	16 (19%)
64	404	–	12 (3%)	1 (< 1%)	–	9 (2%)	370 (92%)	12 (3%)
128	14	–	–	–	–	–	14 (100%)	–
> 128	18	–	–	–	–	–	17 (94%)	1 (6%)
Total number of isolates		16	133	27	14	32	506	472

*ESBL* extended-spectrum β-lactamase, *IMI/REL* imipenem/relebactam, *KPC Klebsiella pneumoniae* carbapenemase, *MBL* metallo-β-lactamase, *MIC* minimum inhibitory concentration, *NS* non-susceptible

^a^Class D enzymes were not detected in any of the isolates collected

^b^Includes SMART 2009, 2011, 2015, and 2016 data. Approximately 100 isolates possessed more than one acquired β-lactamase. For purposes of this table, each of these isolates was only counted once; the specific category each isolate was assigned to was based on the following algorithm: MBL > KPC > GES > other ESBL

^c^Imipenem minimum inhibitory concentration in the presence of 4 μg/mL of relebactam

^d^Isolates that only encoded AmpC, and not any of the other studied β-lactamases. All 472 isolates contained a gene for chromosomal PDC and 2 isolates also that contained a gene for a plasmid-borne AmpC (ie, FOX-14 and DHA-1); both of these isolates had an imipenem/relebactam MIC of 8 μg/mL

An exploratory analysis was conducted to see if there were any obvious contributions of PDC sequence to imipenem or IMI/REL susceptibility. Of the 2691 isolates from SMART 2011, 2015, and 2016 that possessed only PDC alleles and no other detected β-lactamase gene, relebactam restored susceptibility to 2283 isolates (85%). The MIC range, MIC_50_, and MIC_90_ for imipenem and IMI/REL by PDC allele are shown in the online supplement (Additional file 3: Table S4). No relationship was observed between PDC alleles and either imipenem (Additional file 4: Figure S1A) or IMI/REL MIC values (Additional file 4: Figure S1B). The majority of PDC alleles had alanine at position 105, and only 3 (PDC-1, − 6, and − 98) had threonine. MIC_50_ and MIC_90_ values for IMI and IMI/REL were mostly similar regardless of the residue at this location.

## Discussion

These in vitro studies using surveillance and challenge panels demonstrate that the novel BLI relebactam, when combined with the carbapenem imipenem, has the potential to overcome key mechanisms of imipenem resistance in *P. aeruginosa* and could thus restore imipenem susceptibility against many MDR strains of this pathogen. Relebactam was found to have no intrinsic activity against *P. aeruginosa* and no inoculum effect. Enzymology studies showed relebactam to be a potent, mechanism-based inhibitor of *P. aeruginosa* AmpC, binding to the enzyme with low to sub-μM initial affinity, followed by efficient acylation (on the order of minutes). The acylated enzyme/inhibitor complex was shown to undergo slow intramolecular deacylation, reverting back to the non-covalent enzyme/inhibitor complex and resulting in an overall target engagement time of > 1.5 h. Furthermore, the acylated complex did not appear to undergo appreciable destructive hydrolysis by intermolecular water-mediated deacylation, yielding a turnover number near unity (ie, approaching that of an irreversible inhibitor).

Additional in vitro analyses with > 14,000 isolates showed that 89% of all *P. aeruginosa* isolates were susceptible to the combination of imipenem with relebactam at 4 μg/mL using current Clinical and Laboratory Standards Institute (CLSI) breakpoints for imipenem, including 68% of all isolates that were not susceptible to imipenem alone.

Our studies confirmed the previously reported ability of relebactam to inhibit class C β-lactamases in *P. aeruginosa* (relebactam is also known to inhibit class A β-lactamases) [48]. This is a noteworthy finding, given that overexpression of AmpC (a class C enzyme) in conjunction with decreased membrane permeability to imipenem is an important mechanism conferring imipenem non-susceptibility to *P. aeruginosa* [41–44]. Although some BLI are subject to efflux in *P. aeruginosa* (eg, avibactam) [53], relebactam was purposefully designed to avoid this issue. Our experiments showed that relebactam was not affected by efflux at basal levels of efflux pump expression. Parallel results with relebactam and various analogues suggest that the presence of a positively charged side chain attached to the DABCO core may be the structural basis for avoiding efflux. Relebactam and BLI compound 3, which were not subject to efflux in our experiments, both possess piperidine residues, which would be protonated (and hence, positively charged) at physiological pH. Conversely, BLI compounds 1 and 2 are less basic and would not be protonated, thus possessing a neutral charge at physiological pH.

Furthermore, susceptibility to IMI/REL was also unaffected by RND efflux pump overexpression, demonstrating that relebactam is not a substrate of any key RND efflux pumps. This result is clinically relevant, because such overexpression is often selected as a resistance mechanism to various antibacterial agents (eg, the carbapenem meropenem). In previous genetic studies, imipenem was shown conclusively not to be a substrate for any of the RND efflux pumps in *P. aeruginosa*, unlike the beta-methyl carbapenem meropenem. In *P. aeruginosa*, meropenem is a substrate for mexAB/OprM, mexCD/OprJ, and mexEF/OprN, while there is no effect on imipenem by any combination of RND pumps [54, 55]. This was confirmed by our experiments. However, there can be effects on imipenem MIC values when efflux pump genes are regulated concomitantly with genes whose expression affects imipenem MIC values. For example, an *nfxB* mutation results in overexpression of mexCD/OprJ and an *nfxC* mutation in overexpression of mexEF/OprN. Greater susceptibility to imipenem in *nfxB* mutants was found to be a result of concomitant reduction in AmpC expression [52]. Increased imipenem MIC values in *nfxC* mutants are correlated with the action of the MexT activator via a concomitant reduction in OprD expression, rather than the overexpression of mexEF/OprN [56].

AmpC-mediated resistance to imipenem in *P. aeruginosa* is multi-factorial, depending on both loss of the outer membrane entry porin OprD and high-level expression of the chromosomally-encoded AmpC enzyme. Neither OprD porin loss in the absence of AmpC induction or derepression, nor AmpC constitutive expression without concomitant loss of the OprD porin will result in imipenem MIC values over the susceptibility breakpoint [57]. However, AmpC expression influences the imipenem MIC even in OprD-proficient isolates, as evidenced by the reduction in imipenem MIC in the presence of relebactam. This phenocopies genetic results seen with an isogenic strain set of *P. aeruginosa* where imipenem MIC values varied upon AmpC overexpression or deletion within the context of OprD proficiency or deficiency [57]. Imipenem is an inducer of AmpC, but is fairly stable to its hydrolysis, which explains why frank resistance does not occur until both entry of imipenem is limited and enzymatic activity is enhanced by mass action [58].

Since neither imipenem nor relebactam are subject to efflux, inhibition of the chromosomal AmpC enzyme by relebactam will restore susceptibility to many MDR isolates of *P. aeruginosa*, including those with overexpression of efflux pumps, as long as these isolates do not possess β-lactamases not inhibited by relebactam (eg, MBLs, some GES enzymes, and some class D carbapenemases). Overall, the inhibition of AmpC by relebactam in vitro was in agreement with the restoration of imipenem activity against imipenem-resistant strains of *P. aeruginosa* seen in our analyses. However, some isolates producing only a chromosomally encoded AmpC enzyme were not susceptible to the combination. Because PDC alleles did not impact imipenem and IMI/REL MIC values, it is likely that these isolates expressed a particularly high amount of PDC, thus influencing the ability of relebactam to restore imipenem susceptibility in these isolates, as reported previously [59]. It is also possible that some isolates could express unknown/undetected β-lactamases or imipenem resistance mechanisms. In addition, a recent analysis showed that the GES enzyme may also contribute to non-susceptibility to the combination, and attributed the resistance to IMI/REL to the expression of both GES and OXA in 2 *P. aeruginosa* clinical isolates [60]. In our study, GES enzymes were seen among both IMI/REL susceptible and NS isolates. The amino acid at position 170 has been implicated as important for the spectrum of hydrolysis by GES [61]. We observed a diversity of GES enzymes with ESBL or carbapenemase characteristics across susceptible and NS isolates.

No influence of the sequence of PDC was observed among the clinical isolates examined. While Rodriguez-Martinez and colleagues observed that a mutation of the amino acid at position 105 from threonine to alanine increased the MIC to imipenem alone, it should be noted that this observation was made in isogenic strains of *P. aeruginosa*, and clinical susceptibility also depends on expression level [62]. In the present study, of the 2410 isolates examined with more than 10 representatives of any one PDC allele, only 3 alleles (PDC-1, − 6, and − 98; *n* = 335) possessed threonine at position 105, whereas the vast majority possessed the T105A substitution, and the MIC_50/90_ and MIC range were not any higher than those alleles with alanine at position 105.

Other novel BLI approved or in late-stage clinical development include avibactam and vaborbactam. It is important to understand how these compounds differ in their activity against *Pseudomonas spp.* when combined with various β-lactam partners, and our data are useful in furthering this understanding. Similar to relebactam, avibactam is a DABCO inhibitor that potently inhibits class A and class C enzymes; previously presented data suggest that intact avibactam is recreated after initial binding to a susceptible β-lactamase and thus able to inhibit additional β-lactamase molecules [63]. We observed the same with relebactam in our enzymology studies (Fig. 1), but for both avibactam and relebactam the reaction time scale is probably too long for this effect to be biologically and clinically meaningful. Avibactam has been approved for the treatment of gram-negative infections in combination with the third-generation, antipseudomonal cephalosporin ceftazidime, and currently also is being investigated in combination with aztreonam. While aztreonam-avibactam likely has no utility in the treatment of *P. aeruginosa* [64], ceftazidime-avibactam is already approved for this indication, with recent surveillance showing > 95% of *P. aeruginosa* isolates to be susceptible [13, 30, 65]. On the other hand, some investigators have reported up to 40% resistance to ceftazidime-avibactam in MDR *P. aeruginosa* [66], and another cephalosporin-BLI combination (ie, ceftolozane-tazobactam) has been shown to be more active against carbapenem-resistant *P. aeruginosa* in vitro [67, 68]. Ceftolozane-tazobactam may therefore be preferable to ceftazidime-avibactam for treating MDR *P. aeruginosa* [69]. The carbapenem-BLI combination of meropenem-vaborbactam does not appear to have improved in vitro activity against *P. aeruginosa* compared with its carbapenem component alone [70]. It can therefore be expected that meropenem-vaborbactam would possess only limited clinical utility in the treatment of MDR *P. aeruginosa*.

Thus far, the combination of imipenem and relebactam has shown promising results in both phase 2 and phase 3 clinical studies in complicated urinary tract infection and complicated intra-abdominal infection, including among patients with *P. aeruginosa* infections [71–73]. However, both phase 2 studies broadly enrolled patients regardless of baseline pathogen susceptibility profiles and were not limited to those with MDR causative pathogens. The clinical efficacy of IMI/REL was supported by a recent phase 3 study in patients with imipenem-resistant bacterial infection [73], and further confirmation is sought from another phase 3 study in patients with bacterial pneumonia that was recently completed (NCT02493764).

## Conclusions

Relebactam was found to be a potent, slowly dissociated inhibitor of *P. aeruginosa* AmpC, with a practical effectiveness of inhibition approaching that of an irreversible inactivator. Almost 90% of all *P. aeruginosa* isolates analyzed (> 14,000) were susceptible to the combination of imipenem with 4 μg/mL relebactam, including about 70% of all imipenem-NS isolates. We identified specific properties of relebactam (ie, the lack of an inoculum effect and the lack of efflux) that likely contribute to the in vitro efficacy of this agent in restoring imipenem susceptibility. In our data set, relebactam was particularly effective in *P. aeruginosa* strains encoding AmpC (class C) β-lactamases, regardless of the underlying PDC allele. Conversely, isolates not susceptible to IMI/REL at current imipenem breakpoints frequently contained class B MBLs. The combination of imipenem with relebactam may therefore have potential in the treatment of carbapenem-resistant *P. aeruginosa* infections, with the exception of strains carrying class B or class D carbapenemases.

## Methods

### Enzymology studies

#### Determination of AmpC inhibition kinetic parameters

AmpC PDC-1 was monitored in a continuous homogeneous biochemical assay via hydrolysis of a colorimetric nitrocefin substrate in 384-well plates (for PDC-1 cloning and purification, see Additional file 1); the assay buffer used was 0.1 M KH_2_PO_4_, pH 7.0 with 0.005% Tween 20. Relebactam (in concentrations ranging from 0.61 nM to 20,000 nM) and 200 μM nitrocefin were dispensed first and AmpC (30 pM) was subsequently added to start the reaction. The absorbance increase at 490 nm was immediately and continuously monitored for 2 h. Inactivation kinetic parameters were determined by global progress curve analysis of the entire kinetic data set using a validated and previously published experimental design (Additional file 2: Figure S1) [74–76].

#### Determination of relebactam turnover number

*P. aeruginosa* AmpC (PDC-1) and serially diluted relebactam were pre-incubated at 200× final concentrations (300 pM *P. aeruginosa* AmpC and 14.7 pM to 30,000 pM relebactam) for 2 and 24 h and subsequently diluted to 1× concentrations and assayed under saturating nitrocefin concentration (200 μM). The residual (ie, quickly recoverable) enzymatic activity was monitored continuously by absorbance measurement at 490 nm for 10 min. The turnover number was determined by extrapolation to 0% activity from plots of the initial linear portion of % activity versus inhibitor/enzyme ratio.

### Isolate sources

Unique clinical isolates of *P. aeruginosa* were obtained from 4 different sources, with no overlap: (a) a challenge panel of imipenem-NS isolates (*n* = 108) curated at Merck & Co., Inc. (Kenilworth, NJ, USA) [77]; (b) a challenge panel of imipenem-NS isolates (*n* = 185) curated at Eurofins (Chantilly, VA, USA) [78]; (c) the Study for Monitoring Antimicrobial Resistance Trends (SMART) global surveillance program in the years 2009, 2011, 2015, and 2016 (*n* = 14,813 total isolates, including 4501 imipenem-NS isolates as of October 2018) [10]; and (d) all imipenem-NS isolates from a previously published surveillance study from New York, NY, USA (*n* = 144) [45]. Details of the 4 isolate sources are found in the online supplement (Additional file 3: Table S1). These isolate sources were chosen to provide a broad distribution of geographic areas and imipenem MIC values.

#### Molecular epidemiology

Imipenem-NS isolates from SMART 2009, 2011, 2015, and 2016 were analyzed for the presence of the following β-lactamases by an established method using multiplex polymerase chain reaction (PCR) followed by full-gene DNA sequencing [79–81]: class A (SHV, TEM, CTX-M, VEB, PER, GES, and KPC); class B (GIM, IMP, NDM, SPM, and VIM); class C (acquired, plasmid-mediated AmpC: ACC, ACT, CMY, DHA, FOX, MIR, and MOX; and intrinsic, chromosomal AmpC: PDC); and class D (OXA-24/− 40–like). PDC sequences [82] were retrieved from Genbank by searching the protein database for the keyword “PDC-ˮ. Retrieved sequences were aligned with the Clustal Omega software (EMBL-EBI, Hinxton, United Kingdom) [83] using the default protein settings. The potential relationships of imipenem and IMI/REL MIC values to PDC sequence were explored by determining the MIC_50_ and MIC_90_ for all isolates possessing only a PDC allele and no other detected β-lactamase; the MIC_50_ and MIC_90_ values associated with PDC alleles present in ≥10 isolates were mapped onto a circular dendrogram. The resulting dendrogram was visualized using the Dendroscope 3 software (Universität Tübingen, Tübingen, Germany) [84]. Molecular data were available for all imipenem-NS isolates collected in 2015 and 2016. For 2009, selected imipenem-NS isolates underwent molecular characterization. For 2011, only isolates NS to the combination of imipenem and relebactam 4 μg/mL underwent molecular characterization.

### Relebactam efflux studies

#### Efflux studies using isogenic mutant strains

To assess whether relebactam and 3 additional DABCO analogs chosen for illustrative purposes were subject to efflux, we isolated *oprD*^−^ mutants in an isogenic set of *P. aeruginosa* strains: (a) a wild-type strain (CB046, also known as MB5919) expressing baseline levels of efflux pumps and (b) a mutant strain (CB1101, also known as MB5890) with multiple deletions of genes encoding major RND efflux pump proteins (Δ[mexAB-oprM] Δ[mexCD-oprJ] Δ[mexXY] Δ[mexJKL] Δ[mexHI-opmD] Δ[opmH]) [85, 86]. Of note, it was later determined that the background of this strain pair carried an 8-base pair deletion in *mexT*, which has previously been described as an *nfxC*-type mutation that in other *P. aeruginosa* strains results in overexpression of the MexEF-OprN efflux pump and repressed OprD porin production, and raised the imipenem MIC slightly [56, 87]. The *oprD*^−^ mutants of both strains were obtained by selection for spontaneous imipenem resistance on agar plates containing concentrations of imipenem above the MIC, using a previously described method [88]. Gel electrophoresis of outer membrane proteins was used to confirm loss of the band corresponding to OprD. The resulting mutant strains, MB6477 (OprD^−^, wild-type efflux-proficient) and MB6476 (OprD^−^, multi-efflux–deficient), were then used to determine if these BLI were subject to efflux by calculating the fold differential between the concentrations required in broth microdilution testing to reduce the imipenem MIC to 4 μg/mL, expressed as the BLI concentration required for MB6477 divided by the BLI concentration required for MB6476 for each BLI compound tested.

#### Construction and antibiogram evaluation of *P. aeruginosa* RND overexpression strains

A second set of efflux studies was conducted to determine if susceptibility to relebactam might be affected by multidrug efflux pump overexpression, a common resistance mechanism in MDR *P. aeruginosa*. Four different strains, each overexpressing one of the major RND efflux pumps (ie, MexAB, MexCD, MexEF, and MexXY), were constructed from MB6477 by knocking out the transcriptional regulators *mexR*, *nfxB*, *mexL*, and *mexZ*, respectively. Primer sequences for these experiments are listed in the online supplement (Additional file 3: Table S2). Flanking regions of each gene comprising 500 bp upstream and 500 bp downstream were amplified using primer pairs P1/P2 and P3/P4 for *mexR*, P5/P6 and P7/P8 for *nfxB*, P9/P10 and P11/P12 for *mexL*, and P13/P14 and P15/P16 for *mexZ*. Subsequently, splicing by overlap extension was used to combine upstream and downstream products with external primers P1/P4, P5/P8, P9/P12, and P13/P16 for *mexR*, *nfxB*, *mexL*, and *mexZ*, respectively. These complete alleles containing unmarked deletions of each transcriptional regulator were then cloned into a BamHI/PstI-digested pEX18Ap suicide vector using In-Fusion HD enzyme (Clontech, Mountain View, CA). After DNA sequencing (performed by Genewiz Inc., South Plainfield, NJ) to confirm the correct alleles had been made, plasmids were mobilized from *E. coli* RHO3 into MB5919 and MB6477 using previously published methods [89]. To resolve cointegrants to double crossover mutants, single colonies were restreaked onto lysogeny no-salt broth (10 g/L tryptone, 5 g/L yeast extract) with 10% (wt/vol) sucrose to select for loss of the *sacB*-containing vector backbone. Resulting colonies were screened by PCR using primers P17/P18, P19/P20, P21/P22, and P23/P24 for *mexR*, *nfxB*, *mexL*, and *mexZ*, respectively, to identify those with an amplified DNA product that was reduced in size compared to the wild-type parental strains. Constructs containing the desired deletion were confirmed by sequencing across the respective gene-yielding strains MB5919∆*mexR*, MB5919∆*nfxB*, MB5919∆*mexL*, MB5919∆*mexZ*, MB6477∆*mexR*, MB6477∆*nfxB*, MB6477∆*mexL*, and MB6477∆*mexZ*. In these experiments, all DNA was purified using the QIAprep spin miniprep kit and QIAquick gel-extraction kit (Qiagen, Valencia, CA). Phusion high-fidelity polymerase and restriction enzymes PstI and BamHI were from New England BioLabs (Ipswich, MA). Electroporation was performed on a Gene Pulser II electroporator with Gene Pulser cuvettes (Bio-Rad, Hercules, CA).

Antibiograms, determined by 2-fold serial broth microdilution, were obtained for each of the 4 RND overexpression strains, as well as for the isogenic mutant strains MB6477 and MB6476, using aztreonam, ceftazidime, chloramphenicol, levofloxacin, and meropenem, which are all known to be affected by efflux in *P. aeruginosa* to varying degrees [49, 50], and which served as controls to confirm that the engineered strains were efflux overexpressors. After the phenotypes of efflux overexpression were confirmed, imipenem and imipenem plus 4 μg/mL relebactam were tested subsequently to see if the MICs were affected by efflux.

### Microbiology studies

Antibacterial susceptibility testing for each *P. aeruginosa* isolate obtained from the four isolate sources listed above was performed using standard CLSI broth microdilution methodology using cation-adjusted Mueller Hinton broth [90, 91]. Imipenem MIC values were assessed in both presence and absence of relebactam at a fixed concentration of 4 μg/mL in combination with 2-fold dilutions of imipenem. The in vitro susceptibility concentration of 4 μg/mL relebactam was chosen based on an average total plasma concentration of 4.94 μg/mL relebactam (free concentration = 3.85 μg/mL) achieved in adults (with bodyweight > 70 kg) receiving 250 mg relebactam every 6 h [71]. MIC values for both imipenem and the IMI/REL combination were interpreted using current CLSI breakpoints for imipenem against *P. aeruginosa*: susceptible, 2 μg/mL; intermediate, 4 μg/mL; and resistant, 8 μg/mL [91].

Relebactam was assessed for intrinsic antibacterial activity in the Merck challenge panel (*n* = 108 isolates) by testing in 2-fold serial dilutions ranging from 0.06 μg/mL to 64 μg/mL. The potential for an inoculum effect of relebactam plus imipenem was evaluated by testing in duplicate the MIC values of imipenem alone and imipenem plus relebactam 4 μg/mL in 3 strains of *P. aeruginosa:* (a) ATCC 27853, an imipenem-susceptible, standard reference strain of *P. aeruginosa* expressing PDC-5; (b) CL 5701 (M1405β-con D2–4105), an imipenem-NS strain with de-repressed AmpC (PDC-5) expression and spontaneous loss of OprD [57]; and (c) CLB 24228, an imipenem-NS strain with inducible AmpC expression (PDC-16). Each strain was tested at the standard inoculum of 5 × 10^5^ colony forming units (CFU)/mL, as well as at inocula lower (5 × 10^4^) and greater (5 × 10^6^) than the standard inoculum.

## Additional files

Additional file 1: PDC-1 cloning and purification. (DOCX 13 kb)

Additional file 2: **Figure S1.** Mechanistic profiling by global progress curve analysis. (DOCX 81 kb)

Additional file 3: **Table S1.** *P aeruginosa* isolate sources. **Table S2.** Primers used in the construction of *P. aeruginosa* RND-overexpressing strains. **Table S3.** Inoculum effect control data. **Table S4.** Susceptibility to imipenem and the combination of imipenem with relebactam 4 μg/mL by PDC allele. (DOCX 25 kb)

Additional file 4: **Figure S1.** Minimum inhibitory concentrations (MIC_50/90_, expressed in μg/mL) for imipenem (A) and imipenem with 4 μg/mL relebactam (B), mapped onto a dendrogram of *P. aeruginosa* PDC alleles. (PDF 1384 kb)

## Data Availability

The dataset(s) supporting the conclusions of this article are included within the article and its additional files. The data sharing policy of MSD, including restrictions, is available at http://engagezone.msd.com/ds_documentation.php. Requests for access to the data for this study can be submitted through the EngageZone site or via email to dataaccess@merck.com.

## Abbreviations

BLI: β-lactamase inhibitor
bp: Base pair
CFU: Colony forming units
CLSI: Clinical and Laboratory Standards Institute
DABCO: Diazabicyclooctane
IMI/REL: Imipenem/relebactam
KPC: *Klebsiella pneumoniae* carbapenemase
MBL: Metallo-β-lactamase
MDR: Multidrug-resistant
MIC: Minimum inhibitory concentration
MK-7655: Relebactam
NS: Non-susceptible
OprD: Outer membrane porin D
PBP: Penicillin-binding protein
PCR: Polymerase chain reaction
PDC: *Pseudomonas*-derived cephalosporinase
RND: Resistance-nodulation-cell division
XDR: Extensively drug-resistant
