# Supplementary material for: In vitro studies evaluating the activity of imipenem in combination with relebactam against Pseudomonas aeruginosa
Source: BMC Microbiol. 2019 Jul 4;19:150. doi: 10.1186/s12866-019-1522-7 (PMC6610938; doi:10.1186/s12866-019-1522-7)
Supplement: Supplementary file 1 — PDC-1 cloning and purification. (DOCX 13 kb) [file 12866_2019_1522_MOESM1_ESM.docx]

**PDC-1 cloning and purification.**

In order to create an expression vector which would allow the N-terminal in-frame addition of glutathione S-transferase (GST) and a thrombin cleavage site, oligonucleotides flanking the GST-thrombin site from pGEX-4T-1 and incorporating a 5' NdeI site and a 3' HindIII site were synthesized. Polymerase chain reaction (PCR) with Platinum Taq HiFi DNA polymerase produced the expected 685 base pair insert for the GST. This fragment was cloned into a TOPO vector (Thermo Fisher Scientific) and the sequence verified. The gene was cloned into NdeI-HindIII sites of the pET26b(+) vector enabling N-terminal GST fusions with a thrombin site for removal of the GST tag. The PCR primers designed for the β-lactamases incorporated a 5'HindIII site and a 3'XhoI site to allow directional cloning into this vector. A modified version of the vector was also made using a HindIII linker which incorporated EcoRI and BamHI sites downstream of the HindIII site to allow other cloning methods.

AmpC (ie, PDC-1) lacking the periplasmic signal sequence was cloned into the modified pET26b(+) vector at the HindIII site.^1^

KPC-2 and *P. aeruginosa* AmpC PDC-1 expression plasmids were isolated from host INVαF´ using standard kits (Thermo Fisher Scientific). The plasmid was then transformed into competent *E. coli* BL21 Star (DE3) cells (Invitrogen) according to the manufacturer's instructions. *E. coli* BL21 Star (DE3) cells containing a plasmid with an IPTG inducible gene were grown overnight on Luria Bertani (LB) agar plates containing kanamycin at 30 mg/mL (LB/Kan). A streak was used to inoculate LB/Kan broth and the culture grown at 37°C with shaking at 250 revolutions per minute (rpm). When the culture reached the optical density at 600 nm, 0.1 mM isopropyl β-D-1-thiogalactopyranoside (IPTG) was added and the cultures were incubated at 30°C 250 rpm. After 4 hours, cultures were terminated by centrifugation at 4400 × g for 15 minutes. The cell pellet was stored at 20°C.

Cell pellets from 2 liters of cells grown as above were thawed, resuspended in buffer A (20 mM sodium phosphate, 150 mM sodium chloride, 5 mM dithiothreitol (DTT), and 10% glycerol, pH 7.4) and lysed with two passes through a French Pressure cell (1250 psi). The 10 mL of lysed material was cleared by centrifugation for 45 minutes at 35,000 × g. Glutathione Sepharose (8.5 mL) was washed with buffer B (50 mM Tris-HCl, 500 mM sodium chloride, 1 mM DTT, 1 mM EDTA, and 10% glycerol, pH 7.4), added to the supernatant, and rocked on a Nutator for 30-60 min at room temperature. The resin was washed with buffer B until the Bio-Rad assay was at background. Thrombin cleavage buffer (9 mL of 1×) containing 64 mL of the biotinylated thrombin was incubated with the 8.5 mL glutathione sepharose for 1 hour at room temperature. Elution of the cleaved material with buffer B resulted in a 10 mL pool. To remove the biotinylated thrombin, 1.5 mL of streptavidin agarose slurry was added and incubated for 30 minutes on the Nutator at room temperature. Following a 500 × g centrifugation for 3 min, the highly purified β-lactamase was recovered.

**Reference cited:**

1. Berrazeg M, Jeannot K, Ntsogo Enguéné VY, Broutin I, Loeffert S, Fournier D, Plésiat P. Mutations in β-Lactamase AmpC Increase Resistance of Pseudomonas aeruginosa Isolates to Antipseudomonal Cephalosporins. *Antimicrob Agents Chemother*. 2015;59(10):6248-55. doi: 10.1128/AAC.00825-15.
