# Supplementary material for: In vitro studies evaluating the activity of imipenem in combination with relebactam against Pseudomonas aeruginosa
Source: BMC Microbiol. 2019 Jul 4;19:150. doi: 10.1186/s12866-019-1522-7 (PMC6610938; doi:10.1186/s12866-019-1522-7)
Supplement: Supplementary file 2 — Figure S1. Mechanistic profiling by global progress curve analysis. (DOCX 81 kb) [file 12866_2019_1522_MOESM2_ESM.docx]

Figure S1. Mechanistic profiling by global progress curve analysis.


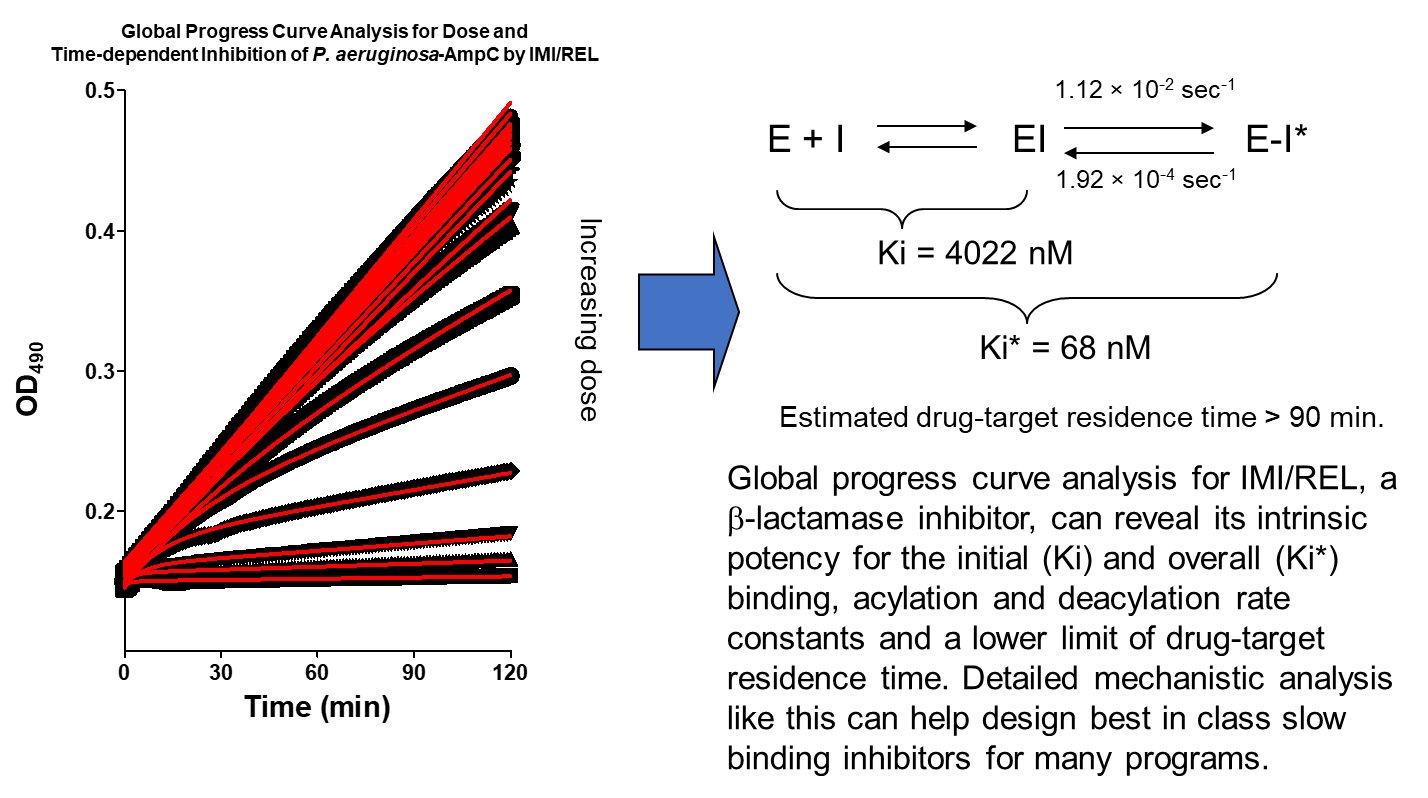


E: AmpC β-lactamase; EI: noncovalent AmpC β-lactamase/relebactam complex; E-I*: acylated AmpC β-lactamase/ relebactam complex; I: relebactam; IMI/REL: imipenem/relebactam; K_i_: dissociation constant for the initial step of noncovalent binding; K_i_*: overall dissociation constant for initial binding and subsequent acylation/deacylation step; OD_490_: optical density at 490 nm.
