# Supplementary material for: In vitro studies evaluating the activity of imipenem in combination with relebactam against Pseudomonas aeruginosa
Source: BMC Microbiol. 2019 Jul 4;19:150. doi: 10.1186/s12866-019-1522-7 (PMC6610938; doi:10.1186/s12866-019-1522-7)
Supplement: Supplementary file 3 — Table S1. P aeruginosa isolate sources. Table S2. Primers used in the construction of P. aeruginosa RND-overexpressing strains. Table S3. Inoculum effect control data. Table S4. Susceptibility to imipenem and the combination of imipenem with relebactam 4 μg/mL by PDC allele. (DOCX 25 kb) [file 12866_2019_1522_MOESM3_ESM.docx]

**Table S1.** *P. aeruginosa* isolate sources.

| ***P. aeruginosa* panel** | **Details** |
| --- | --- |
| Merck challenge (52) | - 108 imipenem-NS isolates - Source: respiratory, urinary tract, wounds, and blood - Majority of isolates collected from 2002 -2006 - Geographic distribution: 78% North America, 16% Europe, 6% Asia |
| Eurofins (53) | - 185 imipenem-NS isolates - Majority of isolates collected in 2009 (75%); rest collected in 2005, 2008, and 2010 - Geographic distribution: 32% Europe, 31% Asia/South Pacific, 29% North America, 8% Latin America |
| SMART global surveillance program (10) | - 14,813 total isolates, including 4501 imipenem-NS isolates^a,b^ - Source: intra-abdominal, urinary tract, and lower respiratory tract - Collected in 2009, 2011, 2015, and 2016 - Geographic distribution: Africa (2-7%), Asia (14-19%), Europe (27-31%), Latin America (12-19%), Middle East (2-7%), North America (21-27%), and South Pacific (4-9%)^c^ |
| Lapuebla et al (45) | - 144 imipenem-NS isolates - Collected between November 2013 and January 2014 - Geographic distribution: 100% North America (11 hospitals in New York City, NY) |

NS: nonsusceptible.

^a^Identification was confirmed using matrix-assisted laser desorption ionization–time of flight spectrometry (Bruker Daltonics, Billerica, MA, USA) in a central microbiology lab (International Health Management Associates, Schaumburg, IL, USA).

^b^Includes SMART surveillance data through October 2018.

^c^SMART isolates from India and China were excluded because molecular data were not collected for these countries.

Table S2. Primers used in the construction of *P. aeruginosa* RND-overexpressing strains.

| **Primer** | **Associated gene** | **Sequence^a^** |
| --- | --- | --- |
| P1 | *mexR* | CCAAGCTTGCATGCCTGCAGtgaggatgatgccgttcacctg |
| P2 | *mexR* | cccaggtcctgcaggttcagcttatcagtcgagtcgctggcaatcgagc |
| P3 | *mexR* | ctcgattgccagcgactcgactgataagctgaacctgcaggacctggg |
| P4 | *mexR* | CGGTACCCGGGGATCCtacggccgcttcaacgacttcg |
| P5 | *nfxB* | CCAAGCTTGCATGCCTGCAGggttccgcgccagctcaccctc |
| P6 | *nfxB* | ccgtggtcctcgagcatctgcttatcagccggccgcctcggccagttc |
| P7 | *nfxB* | gaactggccgaggcggccggctgataagcagatgctcgaggaccacgg |
| P8 | *nfxB* | CGGTACCCGGGGATCCagcagggcgagggcggtgcgc |
| P9 | *mexL* | CCAAGCTTGCATGCCTGCAGctgggaatggctgaccaggttg |
| P10 | *mexL* | gaagtggctgtagacggtgagcttatcagccgttgcagaggaacaggcg |
| P11 | *mexL* | cgcctgttcctctgcaacggctgataagctcaccgtctacagccacttc |
| P12 | *mexL* | CGGTACCCGGGGATCCtcgcgcggctacgcgccgagc |
| P13 | *mexZ* | CCAAGCTTGCATGCCTGCAGcagggcgccgcggctgatgtc |
| P14 | *mexZ* | gacctcgatcttgttcttgtagttatcacatggcagtggtgcccacgcc |
| P15 | *mexZ* | ggcgtgggcaccactgccatgtgataactacaagaacaagatcgaggtc |
| P16 | *mexZ* | CGGTACCCGGGGATCCccgcgcgacccgctcgcggtc |
| P17 | *mexR* | ggccaaaccaatgaactaccccgtgaatcc |
| P18 | *mexR* | tgagcggggcaaacaactcgtcatgcacgc |
| P19 | *nfxB* | acccatcgatgaccctgatttcccatgacg |
| P20 | *nfxB* | aagaaggcgtccagcgcttccaggtaggac |
| P21 | *mexL* | aagcgcatgtcagaatccacctcctccgtc |
| P22 | *mexL* | agaacagctcggaaagcttggggttctgcc |
| P23 | *mexZ* | gaggacgttcagtggccaggaaaaccaaag |
| P24 | *mexZ* | caacagcggctcgttctcgtcgctgcgttc |

RND: resistance-nodulation-cell division.

^a^Transition from upper case to lower case letters denotes the boundary between vector and gene sequence.

Table S3. Inoculum effect control data.

|  |  | **MIC (µg/mL)** | | | | | |
| --- | --- | --- | --- | --- | --- | --- | --- |
|  |  | **Inoculum 5 x 10^5a^** | | **Inoculum 5 x 10^4^** | | **Inoculum 5 x 10^6^** | |
| ***P. aeruginosa* isolate** | **Agent** | **Run 1** | **Run 2** | **Run 1** | **Run 2** | **Run 1** | **Run 2** |
| ATCC 27853 | IMI | 2 | 2 | 2 | 2 | 2 | 2 |
|  | IMI/REL^b^ | 0.5 | 0.5 | 0.5 | 1 | 1 | 1 |
| CL 5701 | IMI | 32 | 16 | 16 | 16 | 32 | 32 |
|  | IMI/REL^b^ | 2 | 2 | 2 | 2 | 4 | 4 |
| CLB 24228 | IMI | 32 | 32 | 32 | 32 | 32 | 32 |
|  | IMI/REL^b^ | 8 | 8 | 4 | 4 | 8 | 8 |

IMI: imipenem; IMI/REL: imipenem/relebactam; MIC: minimum inhibitory concentration.

^a^Standard concentration.

^b^Imipenem tested in combination with relebactam at a fixed concentration of 4 µg/mL.

Table S4. Susceptibility to imipenem and the combination of imipenem with relebactam 4 µg/mL by PDC allele.^a^

|  |  |  | **IMI** | | | **IMI/REL** | | |
| --- | --- | --- | --- | --- | --- | --- | --- | --- |
| **PDC allele** | **N** | **Residue at Position 105** | **MIC range**  **(µg/mL)** | **MIC_50_**  **(µg/mL)** | **MIC_90_**  **(µg/mL)** | **MIC range**  **(µg/mL)** | **MIC_50_**  **(µg/mL)** | **MIC_90_**  **(µg/mL)** |
| PDC-3 | 446 | A | 4->128 | 16 | 32 | 0.25->32 | 2 | 8 |
| PDC-5 | 317 | A | 4-128 | 8 | 16 | 0.25->32 | 1 | 4 |
| PDC-1 | 294 | T | 4->32 | 8 | 32 | 0.25->32 | 1 | 2 |
| PDC-8 | 213 | A | 4->32 | 8 | 16 | 0.25->32 | 1 | 4 |
| PDC-19A | 189 | A | 4-32 | 16 | 16 | 0.5-8 | 1 | 2 |
| PDC-35 | 183 | A | 4-32 | 8 | 16 | 0.5-32 | 2 | 4 |
| PDC-16 | 141 | A | 4->32 | 16 | 32 | 0.25-32 | 2 | 4 |
| PDC-24 | 77 | A | 4->32 | 16 | 32 | 0.25-16 | 2 | 4 |
| PDC-34 | 77 | A | 4->32 | 8 | 16 | 0.25-8 | 1 | 2 |
| PDC-37 | 53 | A | 4->32 | 16 | 16 | 0.5-8 | 1 | 2 |
| PDC-11 | 46 | A | 4-32 | 16 | 16 | 0.25-8 | 2 | 4 |
| PDC-31 | 37 | A | 4->32 | 16 | 32 | 0.25-32 | 1 | 8 |
| PDC-36 | 37 | A | 4->32 | 16 | 32 | 0.25-32 | 2 | 4 |
| PDC-30 | 35 | A | 4->32 | 8 | 32 | 0.25-16 | 1 | 4 |
| PDC-15 | 33 | A | 4->32 | 16 | 16 | 0.25->32 | 1 | 4 |
| PDC-41 | 30 | A | 4-32 | 8 | 16 | 0.5-4 | 1 | 2 |
| PDC-12 | 29 | A | 4-32 | 8 | 16 | 0.25-4 | 1 | 2 |
| PDC-6 | 22 | T | 4->32 | 16 | 32 | 0.25-8 | 2 | 4 |
| PDC-60 | 24 | A | 4-32 | 8 | 16 | 0.5-8 | 1 | 2 |
| PDC-14 | 21 | A | 4-8 | 4 | 8 | 0.25-8 | 2 | 2 |
| PDC-98 | 19 | T | 4-8 | 8 | 8 | 0.5-4 | 1 | 2 |
| PDC-39 | 16 | A | 4-32 | 16 | 32 | 0.5-2 | 1 | 2 |
| PDC-100 | 14 | A | 4-16 | 8 | 16 | 0.5-2 | 1 | 2 |
| PDC-59 | 14 | A | 4-32 | 8 | 16 | 0.12-4 | 1 | 2 |
| PDC-109 | 13 | A | 4-32 | 8 | 32 | 0.25-4 | 1 | 2 |
| PDC-46 | 13 | A | 4->32 | 16 | 32 | 0.5-16 | 2 | 8 |
| PDC-45 | 11 | A | 16 | 16 | 16 | 1-2 | 1 | 2 |

A: alanine; IMI: imipenem; IMI/REL: imipenem/relebactam; MIC: minimum inhibitory concentration; PDC: Pseudomonas-derived cephalosporinase; T: threonine.

^a^In total, 2688 *P. aeruginosa* isolates were evaluated for this analysis. Of these, 2404 isolates (89.4%) expressed a PDC allele that was found in ≥10 isolates; these PDC alleles are included in the table. In an additional 404 isolates, 134 different PDC alleles were found (1 to 9 isolates per allele); these PDC alleles are not included in the table.
