# Supplementary figures and images for: In vitro studies evaluating the activity of imipenem in combination with relebactam against Pseudomonas aeruginosa
Source: BMC Microbiol. 2019 Jul 4;19:150. doi: 10.1186/s12866-019-1522-7 (PMC6610938; doi:10.1186/s12866-019-1522-7)

A

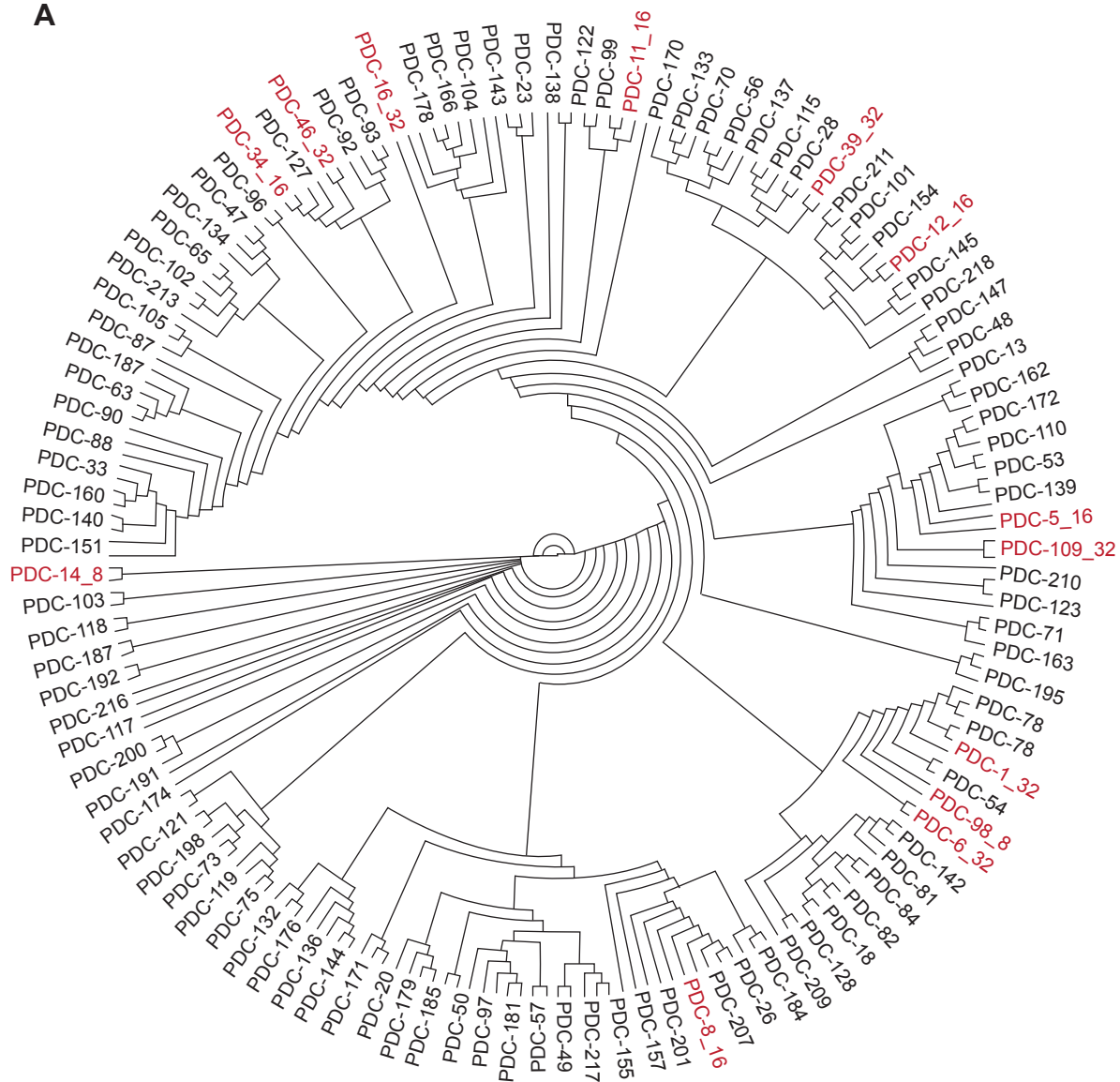

# B

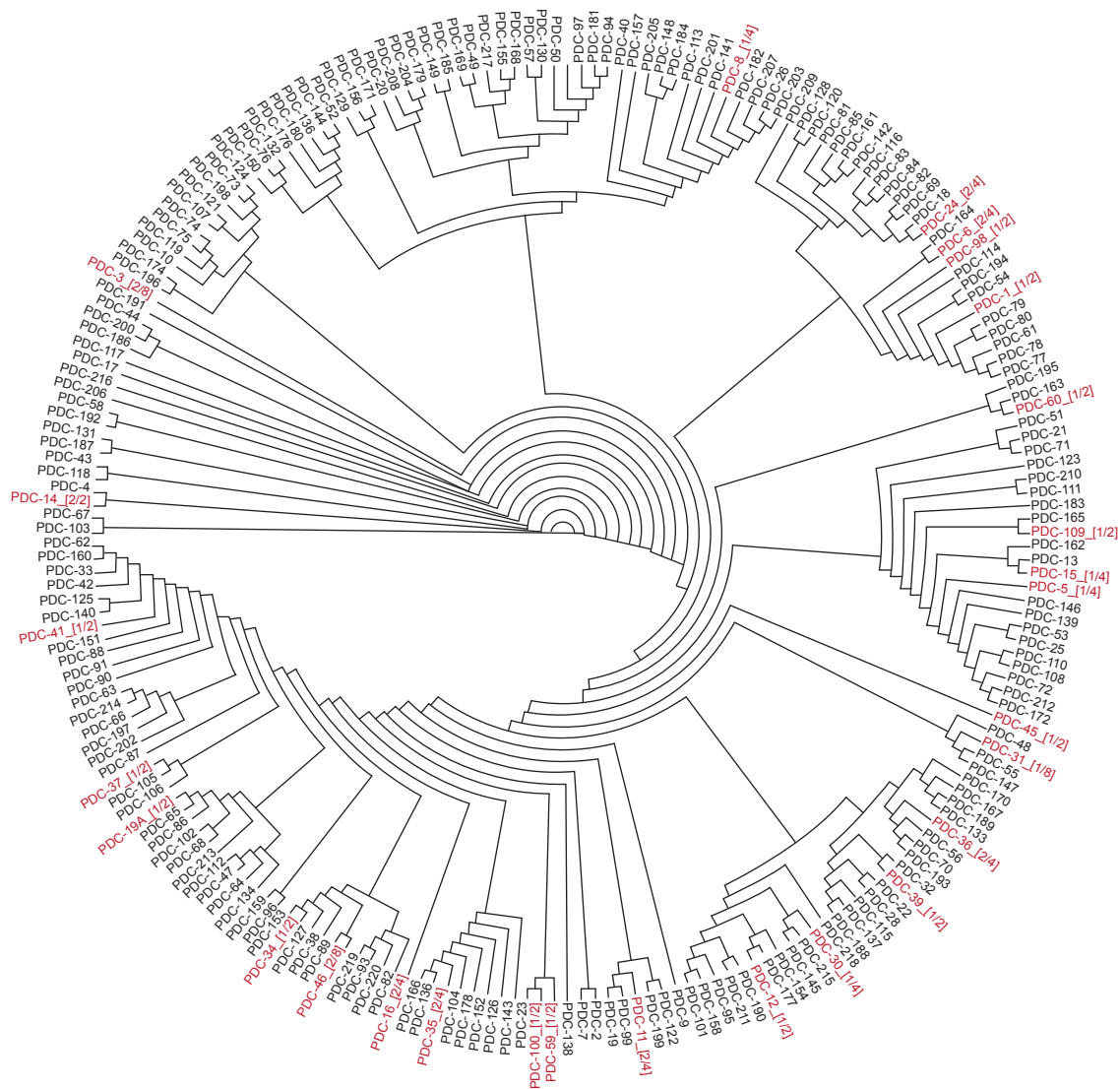

Supplement: Supplementary file 4 — Figure S1. Minimum inhibitory concentrations (MIC50/90, expressed in μg/mL) for imipenem (A) and imipenem with 4 μg/mL relebactam (B), mapped onto a dendrogram of P. aeruginosa PDC alleles. (PDF 1384 kb) [file 12866_2019_1522_MOESM4_ESM.pdf]
